# Early Investigations and Recent Advances in Intraperitoneal Immunotherapy for Peritoneal Metastasis

**DOI:** 10.3390/vaccines6030054

**Published:** 2018-08-10

**Authors:** Anusha Thadi, Marian Khalili, William F. Morano, Scott D. Richard, Steven C. Katz, Wilbur B. Bowne

**Affiliations:** 1 Department of Surgery, Drexel University College of Medicine, Philadelphia, PA 19102, USA; anusha.thadi@gmail.com (A.T.); mariankhalili@gmail.com (M.K.); morano.william@gmail.com (W.F.M.); 2 Department of Obstetrics and Gynecology, Thomas Jefferson University Hospital, Philadelphia, PA 19107, USA; scott.richard@jefferson.edu; 3 Department of Surgery, Boston University School of Medicine, Boston, MA 02118, USA; skatz@chartercare.org

**Keywords:** intraperitoneal immunotherapy, vaccines, CAR-T cells, ascites, carcinoembryonic antigen, folate receptor α, dendritic cells, peritoneal, metastasis

## Abstract

Peritoneal metastasis (PM) is an advanced stage malignancy largely refractory to modern therapy. Intraperitoneal (IP) immunotherapy offers a novel approach for the control of regional disease of the peritoneal cavity by breaking immune tolerance. These strategies include heightening T-cell response and vaccine induction of anti-cancer memory against tumor-associated antigens. Early investigations with chimeric antigen receptor T cells (CAR-T cells), vaccine-based therapies, dendritic cells (DCs) in combination with pro-inflammatory cytokines and natural killer cells (NKs), adoptive cell transfer, and immune checkpoint inhibitors represent significant advances in the treatment of PM. IP delivery of CAR-T cells has shown demonstrable suppression of tumors expressing carcinoembryonic antigen. This response was enhanced when IP injected CAR-T cells were combined with anti-PD-L1 or anti-Gr1. Similarly, CAR-T cells against folate receptor α expressing tumors improved T-cell tumor localization and survival when combined with CD137 co-stimulatory signaling. Moreover, IP immunotherapy with catumaxomab, a trifunctional antibody approved in Europe, targets epithelial cell adhesion molecule (EpCAM) and has shown considerable promise with control of malignant ascites. Herein, we discuss immunologic approaches under investigation for treatment of PM.

## 1. Introduction

**Figure 1:**
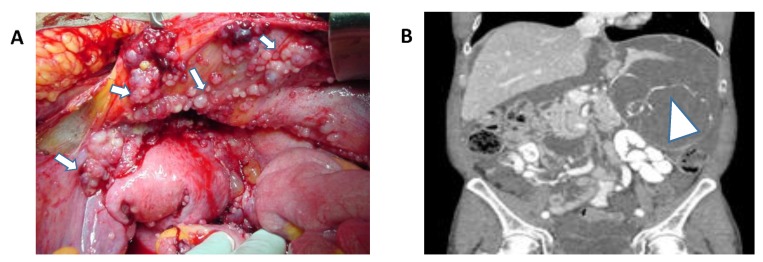
(**A**) Abdominal peritoneal metastasis from ovarian cancer. White arrows demonstrate peritoneal disease (Permission granted by Scott D Richard). (**B**) Coronal CT-scan of the abdomen and pelvis depicting extensive peritoneal metastasis and tumor burden from appendiceal cancer. White arrow demonstrates extensive intraperitoneal disease.

Peritoneal surface malignancies and peritoneal metastasis (PM) develop from a broad spectrum of primary and metastatic malignancies [1,2]. First described in 1899, PM typically leads to symptoms of malignant obstruction and significant tumor burden leaving palliative treatment options and dismal patient outcomes, not exceeding 2 to 6 months (Figure 1) [2,3,4,5,6]. Treatment with systemic chemotherapy, although improving patient outcomes, leads to survival rates ranging between 6.4–17.9 months [2]. Throughout history to current practice, peritoneal disease recurrence after surgery remains high [7,8], resulting in treatment strategies that advocate for aggressive surgical approaches such as tumor debulking and cytoreduction combined with perioperative systemic and intraperitoneal (IP) chemotherapy [9,10,11,12].

Improved survival of cancer patients with PM treated with heated IP chemotherapy (HIPEC) was previously demonstrated in clinical trials conducted in the 1980s [10,11,12]. Recently, the potential benefit of combined cytoreduction and HIPEC was shown by Verwaal and colleagues in patients with colorectal peritoneal metastasis. They cited nearly doubling of 2 year survival rates beyond standard therapy [13,14,15]. However, for colorectal and other peritoneal surface malignancies, the majority of clinical studies are plagued by continued high rates of peritoneal recurrence and poor patient survival [16,17,18]. The burgeoning use of immunotherapy in the management of metastatic malignancies has led to investigations into translational applications for both primary and metastatic regional peritoneal disease [1].

## 2. Immunotherapeutic Breakthroughs in the Modern Era

Several breakthroughs led to historic achievements in the field of immunotherapy. Notably, the concept of immunosurveillance, by Burnet and Thomas, proposing the immune system’s ability to recognize cancer cells that could prevent the development of cancer and multifactorial determinants of ‘tumor immunity’ [19,20]. This understanding led to innovative immunotherapeutic approaches to trigger anticancer immune responses [21]. Subsequent advancements largely evolved from Lloyd J. Old‘s contributions and seminal discovery of the tuberculosis Bacille Calmette-Guerin (BCG) vaccine and tumor necrosis factor [22,23], and Thierry Boon’s observation of immune recognition by T-cells of mutagen-altered tumor antigens [24]. Alan N. Houghton cloned melanoma differentiation antigen-tyrosinase, identified tyrosinase-related protein-1 as the target for monoclonal antibody TA-99, and developed xenogeneic orthologs of self-antigens, ‘altered self,’ which is now widely recognized as a means of breaking immune ignorance and tolerance to poorly immunogenic targets on cancer cells [25,26,27,28,29,30,31,32,33]. Pioneering work by Ralph M. Steinman led to the discovery and central role of the dendritic cell with its inherent ability to present antigens to T cells, acting as an important accessory cell in the development of antigen-specific immune responses [34,35]. 

Immunotherapeutic investigations by Steven A. Rosenberg brought further discoveries utilizing lymphokine-activated killer cells and interleukin 2 (IL-2) , and genetically modified T-cells encoding receptors specific for tumor antigens. Rosenberg’s contributions would later become the foundation of treatment for melanoma and other metastatic malignancies [36,37,38,39]. Further advances include the discovery of cytotoxic T-lymphocyte-associated protein 4 (CTLA-4) by James P. Allison and colleagues’ [40,41], and Gordon J. Freeman’s description of a second pathway of immune inhibition, the programmed death-1 receptor (PD-1) [42,43]. Immune checkpoint inhibitors blocking these pathways led to downregulation of T-cell activation tested as monotherapy and polytherapy for melanoma, renal cell carcinoma and lung cancer [44].

## 3. CAR-T Cell Immunotherapy

CAR-T technology developed by Carl H. June, redirects patient-derived T cells engineered for cancer antigen-specific targeting, with minimal systemic toxicity [45]. Success of CAR-T technology in clinical trials for hematologic malignancies has led to further investigation of its role in treatment of refractory stage IV solid tumors. The main component of a CAR-T cell is an extracellular single chain variable fragment (scFv) from antibodies specific to the cancer antigen of interest and T-cell activating domain. The scFv component of CAR-T cells confers an advantage over specific T-cell therapy, designating CAR-Ts MHC independent [46,47]. A means to induce tumor immunity has been demonstrated using CAR-T cells for solid tumor malignancies. Carcinoembryonic antigen (CEA) has been a target for the development of CAR-T cells against gastric, colorectal, and breast cancers due to its overexpression in solid tumors when compared to normal cells [48]. This novel treatment paradigm has been applied towards metastatic malignancies evidenced by studies directed against hepatic metastasis from colorectal cancer [49]. It has been shown by Parkhurst and colleagues that anti-CEA CAR-T cells proliferate in response to tumors overexpressing CEA, resulting in IL-2 and IFNγ cytokine secretion and tumor cell lysis. Although remarkable, systemic administration of CAR-T cells was associated with severe toxicity limiting its clinical utility [50]. 

Katz and colleagues found that regional delivery anti-CEA CAR-T cells via hepatic artery infusion, reduced immune-mediated damage to CEA expressing cells by specifically targeting tumor metastasis to the liver [49]. In their phase I study, two cohorts of three patients each received CAR-T infusions, with one group also receiving supplemental IL-2 infusions. Importantly, they reported no grade four or five events after administering maximal CAR-T dosage with a single patient living 54 months following hepatic artery CAR-T infusion. In addition, they found elevated serum IFNγ, closely corresponding to significant decreases in CEA in the IL-2 treatment group. These findings were corroborated with image-guided core biopsies showing targeted tumor necrosis and fibrosis as well as preferential CAR-T cell localization to tumors on immunohistochemistry while normal parenchyma was spared [1,49].

Despite advances in regional delivery of CAR-T cells, challenges remain in the immunotherapeutic application of CAR-T cells due to immunosuppressive pathways inherent to solid tumors. Immunosuppression is driven by high levels of the immune checkpoint molecule programmed cell death-ligand (PD-L) as shown in advanced pancreatic, breast, and ovarian cancer. Similarly, activation of T lymphocyte in malignant ascites from ovarian cancer is further suppressed by the presence of PD1/ PD-L1 or B7/H1, the CD274 pathway, or the T cell immunoglobulin and mucin domain containing 3 (TIM3)/ galectin9 pathway [51,52]. Additionally, immunosuppression can also be driven by granulocyte macrophage colony stimulating factor (GM-CSF) and CD4^+^ Fox3^+^ CD25^+^ T regulatory cells (Tregs) which are upregulated in pancreatic ductal adenocarcinoma. GM-CSF is produced by pancreatic ductal epithelial cells (PDECs) bearing an oncogenic *Kras^G12D^* allele and inhibits anti-cancer activity of CAR-Ts via recruitment and proliferation/maturation of Gr-1+ CD11b+ myeloid derived suppressor cells (MDSCs) in lymphoid organs. MDSCs produce nitric oxide (NO) and deplete arginine in the environment to induce apoptosis of T-cells. Thus, suppression of either GM-CSF or MDSCs in combination with immunotherapy can be a potential strategy to reduce tumor burden in patients with PM [53,54,55].

Another surface antigen widely targeted in ovarian, breast, and colorectal cancers by CAR-T therapy is a glycosylphosphatidylinositol-anchored protein, FRα. FRα is localized to the luminal side of polarized epithelial cells in normal tissue, while it is upregulated in tumor cells, losing its polarization. Thus, FRα not exposed to the circulation in normal tissue is accessible to circulation in the setting of malignancy, allowing for tumor-specific targeting by intravenously delivered CAR-T cells [56]. However, there have been initial setbacks with first generation MOv-19ζ CAR-T cells targeting FRα containing CD3ζ intracellular signaling faltering in clinical trials, due to inconsistent localization to tumor sites [57].

Additionally, the harsh tumor microenvironment (low in oxygen and nutrients) poses a challenge for proliferation and survival of CAR-T cells. Moreover, adverse events such as neurotoxicity, cytokine release syndrome, and tumor lysis syndrome leading to hyperkalemia and hyperuricemia have been reported in clinical trials of CAR-T cell treatment [48]. T cell activation and survival is further jeopardized by glucose and glutamine-depleted tumor microenvironments. Enrichment of CAR-T cells can be optimized by co-stimulatory signals; CD28 which promotes aerobic glycolysis and 4-1BB by enhancing fatty acid oxidation as well as mitochondrial biogenesis. These signals also promote effector memory T cells and prolong CAR-T cell survival in circulation [45,58].

## 4. CAR-T Cell Investigations for Peritoneal Metastasis

**Figure 2:**
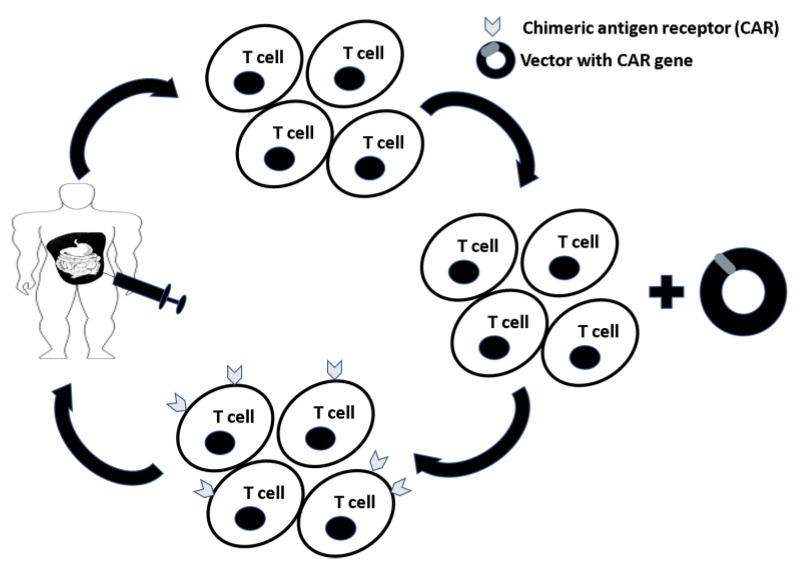
T cells are expanded from peripheral blood mononuclear cells (PBMCs) and transduced with a vector containing the chimeric antigen receptor (CAR) gene. T cells expressing CARs (CAR-T cells) specific for tumor-associated antigens (TAAs) are delivered to the patient intraperitoneally to maximize delivery to the site of disease while minimizing systemic exposure and toxicity.

Advancements in our understanding of the tumor microenvironment has led to developments in CAR-T cell technology with direct intraperitoneal application for treatment of PM (Table 1). T cells expressing chimeric antigen receptor (CAR) gene specific to tumor-associated antigens (TAA’s) are regionally delivered to the peritoneal cavity enhancing CAR-T delivery to the site of the disease avoiding on-target off tumor effects, in addition to mitigating or eliminating cytokine release syndrome and neurotoxicity (Figure 2). We now recognize that the route of CAR-T cell administration significantly impacts tumor localization and regression. Katz et al. introduced regional, hepatic artery infusion of CAR-T cells to treat hepatic tumors from metastatic colorectal cancer [49]. They further investigated the effects of IP vs systemically delivered anti-CEA CAR-T cells in a C57BL6 murine colon adenocarcinoma model. MC38 expressing CEA, which are C57BL6 murine colon adenocarcinoma-derived cells, were cultured with either untransduced or anti-CEA CAR-T cells derived from murine splenic T cells activated by IL-2 prior to culturing. Treatment with CAR-T cells resulted in significant MC38CEA cell lysis as compared to normal splenic T cells. A 37-fold tumor reduction was noted in mice receiving anti-CEA CAR-T cells IP as compared to only three fold tumor reduction in mice receiving anti-CEA CAR-T cells by tail vein injection. This treatment effect was further pronounced when anti-CEA CAR-Ts were delivered in combination with anti-PD-L1 or anti-Gr1 antibodies suppressing MDSCs and Tregs. Furthermore, in response to CAR-T treatment, endogenous T cells shifted to effector memory T cell phenotype (with phenotype CD44+CD62L-CCR7-), which was evident after 28 days as compared to day 10. Moreover, 4 days after IP infusions of anti-CEA CAR-T cells with daily IL-2 injections, a significant increase of systemic IFNγ levels was detected. These preclinical results provide evidence for the potential of combinatory therapy to overcome peritoneal metastasis [59]. Anti-CEA CAR-T cells, given systemically, are now under investigation and currently accruing patients in phase I clinical trials for gastric, colorectal and breast cancer (NCT02349724) [48].

Through further investigations, Song et al*.* demonstrated that regional administration of second generation CAR-T cells promotes long term anti-FRα CAR-T cell persistence and tumor localization. These second generation CAR-T cells were generated by the incorporation of a co-stimulatory signal, CD137, into first generation MOv-19ζ CAR-T cells. CD137 allows for the persistence of memory T cells and CD8^+^ T cells. Furthermore, CD137 promotes expression of BCl-X_L_, which confers resistance to apoptosis and prolongs survival [57]. Han et al*.* incorporated CD137 (4-1BB) in chA21 CAR-T cells to create second generation chA21-4-1BBz CAR-T cells highly specific for cells overexpressing human epidermal growth factor receptor 2 (HER2) (i.e., SKOV3—human ovarian cancer, and NCI-N87—human gastric cancer). In a NOD-SCID mouse xenograft model, treatment with chA21-4-1BBz CAR-T cells resulted in an improved half-life and greater accumulation of CAR-T cells at the tumor site. In addition, second generation CAR-T cell therapy appreciably reduced ascites and tumor burden in this mouse model [60]. Another strategy to increase CAR-T cell localization is the use of endothelin inhibitors to prevent tumor migration [47,57]. 

CAR-T cells have been investigated in ovarian cancer where novel treatment options are sought due to the presence of advanced stages at diagnosis. Koneru et al*.* targeted the extracellular domain of MUC16 (MUC-16^ecto^) which is upregulated in advanced stage ovarian cancer. They constructed anti-MUC-16^ecto^ CAR-T cells co-expressing IL-12 in order to ensure activation and proliferation of these CAR-Ts at the tumor site in the presence of immune checkpoints. These CAR-Ts, when delivered IP in a SCID Beige ovarian cancer xenograft model, were more effective in enhancing tumor reduction and survival in mice as compared to anti- MUC-16^ecto^ CAR-T cells with no IL-12 arm [63].

Another CAR-T platform-derived T cell with chimeric antigen receptor CE7R (CE7^+^R T_CM_) against the CE7 epitope of L1 cell adhesion molecule (L1-CAM) was investigated in a human ovarian cancer (SKOV3) xenograft PM model by Hong et al*.* [61]. This mouse model used IP injections of SKOV3 cells into NOD/scid-IL2Rγnull (NSG) mice, resulting in large volume malignant ascites. L1-CAM was chosen as a potential target for its role in ovarian tumor progression and development of drug resistance. CE7^+^R T_CM_ treatment demonstrated a significant reduction in tumor burden with no detectable ascites. However, T cells against L1-CAM could not prevent tumor recurrence due to the subsequent loss of L1-CAM expression in residual disease. Importantly, combination of CE7^+^R T_CM_ cells with CAR-T cells targeting other antigens may improve the efficiency of this treatment [61,62].

## 5. Cancer Vaccines for Peritoneal Metastasis

**Figure 3:**
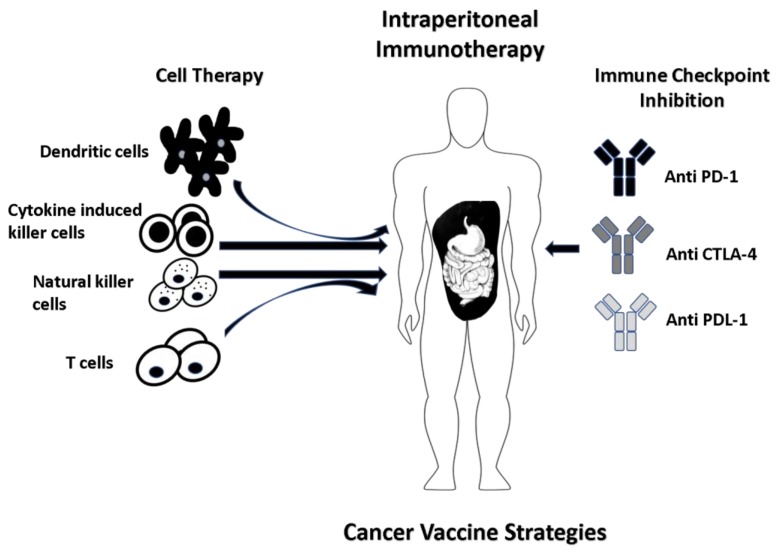
Dendritic cells (DCs) derived from the isolated patient peripheral blood mononuclear cells (PBMCs) are cultured with tumor-associated antigen(s) (TAA) of interest which can be from whole tumor cells, recombinant virus with tumor antigen DNA or peptide-pulsed. The efficiency of anti-tumor activity is further enhanced when DCs are delivered in combination with natural killer cells (NKs), cytokine-induced killer cells (CIKs) and inhibitors of immune checkpoints (anti CTLA-4 and anti PD-1/PDL-1). Another strategy involves in vitro expansion and IP delivery of CD4^+^ T helper cells.

Immunotherapy cancer vaccines have been an area of much interest for decades with significant advances being tailored towards IP treatment of peritoneal metastasis (Table 2 and Figure 3). Malignant ascites poses a major obstacle in evoking an immune response to vaccines. To target ascites, DCs have been combined with cytokine-induced killer cells (CIKs), which are cytotoxic T lymphocytes with CD3^+^ CD56^+^ phenotype. CIKs were chosen for three important reasons: they are minimally cytotoxic towards normal cells, they do not negatively impact hematopoiesis in bone marrow, and they are resistant to apoptosis mediated by Fas ligand. Combination therapy of DCs and CIKs has resulted in the reduction of immunosuppressive Tregs and the accumulation of cytotoxic T cells in ascites, mediated by TNFα and IFNγ [65]. As with CAR-T cells, the mode of administration is of paramount importance in the delivery of cancer vaccines. Natural killer cells (NKs) have proven to be excellent anti-tumor agents, eliciting a strong immune response when combined with DCs. Geller et al. demonstrated that IP delivered NK cells activated by IL-2 improved anti-tumor effects in an ovarian cancer xenograft mouse model compared to systemic delivery [66]. Additionally, Oyer et al. demonstrated that the half-life of IP delivered NK cells in vivo and accumulation at tumor site can be enhanced by PM21 particles which are derived from the plasma membrane of K562-mb21-41BBL cells and membrane bound IL-21 in hematologic malignancies. Moreover, treatment with PM21 resulted in significant activation and proliferation of human NK cells in spleen, lung and bone marrow supporting use as a potential therapy for malignancies in the peritoneal cavity [67,68].

Alkayyal et al*.* later showed the importance of combining pro-inflammatory cytokine IL-12 with oncolytic virus (Maraba MG1) in reducing tumor burden in a peritoneal metastatic colon cancer murine model by inoculation of CT26 (murine colon cancer cells) into Balb/c mice. MG1-IL12-ICV, delivered IP significantly decreased tumor size in these mice, established resistance to re-inoculation by CT26 cells and improved survival. Mechanistically, IL-12 was capable of activating and recruiting NK cells to the tumor site for eradication. These activated NK cells secreted IFNγ, which together with MG1 viral proteins, stimulated DCs leading to further NK cell recruitment [70]. Ma et al*.* assessed the importance of combination of IP delivered antibodies against αCTLA-4 and αPD-L1 with IL-18 in BALB/c mice injected IP with CT26 and 4T1 (mouse breast carcinoma cells). Mice receiving IP infusions of anti-αCTLA-4 and anti-αPD-L1 had a survival advantage over mice receiving tail vein injections. Survival was further prolonged in mice receiving IP antibody therapy along with IL-18 as this cytokine resulted in recruitment of NK and CD8^+^ T cells accompanied with decrease in Tregs in the peritoneal cavity [72,77]. Furthermore, Dobrzanski et al*.* demonstrated that multiple cycles of IP-delivered patient-derived type I CD4^+^ T helper cells (Th1) with cytokines IL-2 and IFNγ, enhanced the anti-tumor activity of autologous CD8^+^ T cells against the tumor-specific glycoform of MUC1 in ovarian cancer patients [75,78].

Malignant ascites leads to poor prognosis and vaccines are being developed and modified to target ascites in an attempt to improve the quality of life in patients with PM. Ai and colleagues evaluated the safety and efficacy of IP-delivered dendritic cell vaccine in combination with CIKs in patients with malignant ascites. This vaccine was well tolerated and resulted in a significant improvement in the quality of life of these patients. IP delivery of this DC vaccine resulted in expansion and cytotoxic activity of CD3^+^ CD56^+^ CIKs and a decrease in Tregs. Moreover, this treatment induced the production of IFNγ capable of blocking angiogenesis and metastasis in cancer cells [65,79].

Reovirus-based anticancer treatment is now an option in patients with PM resistance to current chemotherapies as this method is capable of overcoming immunosuppression by activating DCs and eventually leading to intrinsic anti-tumor T cell activity [74]. A study by Gujar et al*.* employed reovirus-based immunotherapy in a PM murine model developed by injecting female C57BL/6 mice with ID8 (mouse ovarian carcinoma cells). This virotherapy resulted in prolonged survival and delayed PM development in mice along with decrease in Tregs and MDSCs, increase in CD3^+^ and CD8^+^ Tlymphocytes and induction of Th1 cytokine, IFNγ [80]. Reovirus-based therapy has completed clinical trials for various malignancies worldwide including US, UK and Canada. It has completed a phase I study for primary peritoneal cancer and undergone a phase II study for patients with recurrent ovarian and primary peritoneal cancer (NCT01199263, NCT00602277) [81]. Recently, another virus-based immunotherapy, GFP and β glucuronidase in a oncolytic vaccinia virus (GL-ONC1) developed for patients with PM, completed accrual in a non-randomized phase I clinical trial. This vaccinia virus-based vaccine presented a safe profile when delivering IP and successfully established viral infection and replication specifically in ascitic fluid. The vaccine also induced lysis of cancer cells confirmed by the release of GL-ONC1 encoded transgenic β-glucuronidase after oncolysis [69].

Heterogeneity in tumor cells and loss of antigenicity in residual tumors after treatment makes immunotherapy to prevent disease progression and recurrence challenging. In order to address this issue, Chianene-Bullock et al*.* tested the efficacy and tolerability of a multi-peptide vaccine containing five epitopes in patients with ovarian cancer, and fallopian tube and peritoneal cancer. These five epitopes were derived from melanoma differentiation antigen-A1 (MAGE-A1), folate binding protein (FBP) and Her-2/neu which are found in abnormally high levels on ovarian cancer cells. The five epitopes were restricted by human leukocyte antigen (HLA)-A1, A2, A3. Based on this, the vaccine was delivered to patients positive for HLA-A1, A2 and A3 in combination with GM-CSF and montanide ISA-51 adjuvant. Stimulation of T cell activity was detected in eight out of nine patients and the multi-peptide vaccine demonstrated a safe profile in these patients. However, due to poor T cell response, combination of multi-peptide vaccine with immune checkpoint inhibitors and immune modulators should be considered [76].

As previously mentioned, FRα is overexpressed in colon tumors and plays a major role in cancer progression leading to poor survival in patients with PM [82]. A CT26 murine colon cancer model was used by Liang et al*.* to evaluate the anticancer activity of an IP administered FRα targeted liposome-based recombinant IL-15 plasmid (F-PLP/pIL15). Treatment with F-PLP/pIL15 significantly increased the expression of IL-15 in circulation and in ascites leading to substantial tumor reduction via activation of NKs and CD8^+^ T cells. Based on this antitumor efficacy, IL-15 can be a promising cytokine therapy for patients with unresectable PM [71,83]. 

## 6. Catumaxomab

**Figure 4:**
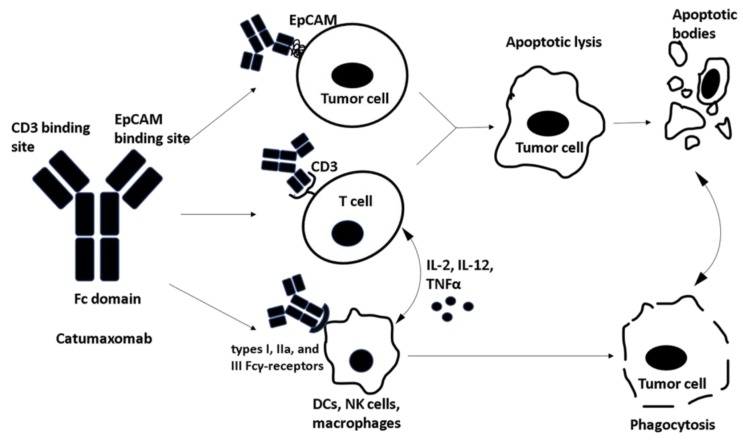
Catumaxomab, a trifunctional antibody contains three important binding sites: One site binds to the epithelial cell adhesion molecule (EpCAM) overexpressed on tumor cells and the second site binds to CD3^+^ T cells. This bispecific antigen binding leads to apoptotic tumor lysis and the resulting apoptotic bodies are phagocytosed. The third binding domain is fragment crystallizable (Fc) which binds to types I, IIa, and III Fcγ-receptors on dendritic cells (DCs), natural killer cells (NKs) and macrophages leading to direct phagocytosis of tumor cells . Furthermore, Fc binding to accessory cells leads to the release of cytotoxic/ pro-apoptotic cytokines.

The trifunctional antibody is an emerging platform capable of producing a long-term vaccination effect. In 2009, catumaxomab became the first drug approved in Europe for treating malignant ascites associated with peritoneal carcinomatosis [84]. IP administration of this bispecific monoclonal antibody has demonstrated a safe profile in clinical trials and is capable of targeting both innate and adaptive immune systems (Table 3). The two antigen-binding sites of catumaxomab target CD3^+^ T-cells and EpCAM receptors while the fragment crystallizable (Fc) domain activates types I, IIa, and III Fcγ-receptors on NK cells. This process leads to phagocytosis of the targeted tumor cells and cell death by pro-apoptotic cytokines such as IL-2, IL-12, and TNF-α [85,86,87] (Figure 4). 

Peritoneal metastasis may be present upon diagnosis in many patients with ovarian cancer, with a large volume of malignant ascites leading to distention and progression of disease. In a study by Burges et al., 23 women with ovarian cancer were treated with catumaxomab for ascites refractory to standard therapy. Treatment with increasing doses of catumaxomab led to a significant reduction in ascites production. Only 1 of the 23 treated patients required paracentesis 28 days after the last infusion, which remains almost 2 weeks longer than typically required [92,94]. In a multicenter study by Wimberger et al., 258 patients with ovarian and non-gynecologic malignancies were randomized to treatment and control groups to determine the effect of catumaxomab therapy on quality of life [90]. Results were determined by patient questionnaires. Treatment with catumaxomab and paracentesis significantly prolonged time to deterioration in quality of life as compared to paracentesis alone. Similarly, a randomized multicenter trial by Heiss et al*.* compared paracentesis alone to paracentesis with post-treatment IP infusion of catumaxomab in the management of malignant ascites from EpCAM^+^ malignancies. This phase II/III clinical trial randomized 258 patients and demonstrated significant improvements in median puncture-free survival and time-to-next-therapeutic-intervention in the experimental group, and improved overall survival in patients with gastric cancer who received catumaxomab [86].

Ströhlein et al. performed a compassionate use study in which nine patients with various peritoneal surface malignancies were treated with escalating doses of catumaxomab via IP infusion [91]. While attempting to improve patient symptoms, the investigators also sought to determine the effect of catumaxomab treatment on the development of long lasting tumor immunity. Tumor cells were harvested during a patient’s initial surgery and were injected sub-dermally 4 weeks after the last catumaxomab treatment. Five of nine patients demonstrated a significant increase in IFNγ producing T cells (0.4% to 2.9%) and maintained these levels as long as 110 days after inoculation in one patient, suggesting long-term immunity. Five of the nine patients also had stable or partial regression of disease, with a mean overall survival of 11.8 months, compared to a mean survival of 6 months in the EVOCAPE study by Sadeghi et al. [91,95]. Catumaxomab has also been shown to have a tolerable toxicity profile in patients with resectable gastric cancer treated perioperatively [88]. Catumaxomab in combination with paracentesis can be effective in treating malignant ascites and improving the overall survival and quality of life of ovarian and gastric cancer patients [91,96].

Overall catumaxomab is well tolerated, with minimal systemic effects when delivered by IP [97]. However, a recent phase I clinical trial demonstrated a dose-dependent hepatotoxicity mediated by the activation of anti-CD3 effector T cells by this trifunctional antibody [89,98]. Activation of these cells led to off-target induction of hepatocytes. Of the 16 patients included in the initial study, one patient suffered fulminant liver failure, leading to cessation of the trial. This study highlights the need for further testing and monitoring of this treatment modality.

## 7. Conclusions

Peritoneal metastasis remains a lethal diagnosis despite modern treatment modalities which may prolong survival. Recent investigations into the use of immunotherapy delivered as intraperitoneal infusion represent a shift in the treatment paradigm for PM, towards treatment as a regional disease rather than systemic metastasis. The use of chimeric T-cells, cell cycle checkpoint inhibitors, tumor-specific antibodies, dendritic cell/tumor antigen vaccines, adoptive autologous T-cells, virus-based vaccines and catumaxomab have shown early promise for management of peritoneal metastasis. As a result, physicians will have more tools in their armamentarium to treat advanced stage malignancies. Clinical studies into the use of intraperitoneal immunotherapy are underway and this treatment strategy may reveal itself as the future treatment paradigm for peritoneal metastasis.

## Figures and Tables

**Table 1:** CAR-T cell therapy for peritoneal metastasis.

Cancer Type	Treatment	Target	Model	Author (Year)
Gastric and Ovarian	chA21-4-1BBz CAR-T cells	HER2	Murine	Han et al. [60] (2018)
Ovarian cancer	CE7^+^R T_CM_ CAR-T cells	L1-CAM	Murine	Hong et al. [61] (2016) Daponte et al. [62] (2008)
Colorectal cancer	Anti CEA CAR-T cells with anti Gr1/GITR and anti PD-L1	CEA, Gr1 and PD-L1	Murine	Katz et al. [59] (2016)
Ovarian cancer	Anti MUC16 CAR-T cells	MUC16	Human	Koneru et al. [63,64] (2015)
Breast and gastric cancer	Anti CEA CAR-T cells	CEA	Human	NCT02349724 (2015)
Ovarian, Breast and Colorectal cancer	Anti FRα CAR-T cells	FRα	Murine	Song et al. [57] (2011)

Abbreviations: PM, peritoneal metastasis; CAR-T, chimeric antigen receptor expressing T cells; CEA, carcinoembryonic antigen; PD-L1, programmed cell death protein-ligand 1; MUC16, mucin 16 associated with membrane; FRα, folate receptor α; HER2, human epidermal growth factor receptor 2; L1-CAM, L1 cell adhesion molecule; NCT, national clinical trial identifier.

**Table 2:** Cancer vaccines for peritoneal metastasis.

Cancer Type	Treatment	Target	Model	Author (Year)
Ovarian cancer, peritoneal carcinomatosis	GL-ONC1	Malignant ascites	Human	Lauer et al. [69] (2018)
Colon cancer	MG1-IL12-ICV	CD69 and IP10	Murine	Alkayyal et al. [70] (2017)
Colon cancer	FRα targeted lipoplex delivering IL-15 gene.	FRα	Murine	Liang et al. [71] (2016)
Colon and breast	Anti PD-L1 and CTLA-4 in combination with IL-18	PD-L1 and CTLA-4	Murine	Ma et al. [72] (2016)
Chronic myelogenous leukemia	NK cells stimulated by IL-21	NKs	Murine	Oyer et al. [68] (2016)
Ovarian cancer, peritoneal metastasis	Survivac vaccine	Survivin	Human	Berinstein et al. [73] (2015)
Colon, ovarian, gastric, pancreatic cancer	Dendritic cell vaccine+CIKs	Tumor inducing cytokines, CD4+CD25+Tregs	Human	Ai et al. [65] (2014)
Ovarian cancer	Reovirus based anti-cancer therapy	Gr 1.1+, CD11b+MDSCs, FOXP3+Tregs, CD3+cells.	Human, Murine	Gujar et al. [74] (2013)
Ovarian cancer	IP delivered human NKs		Murine	Geller et al. [66] (2013)
Ovarian cancer	Anti MUC1 T cells	MUC1	Human	Dobrzanski et al. [75] (2009)
Ovarian cancer	Multipeptide vaccine	MAGE-A1, FBP, Her-2/neu	Human	Chianene-Bullock et al. [76] (2008)

Abbreviations: MDSCs, myeloid derived suppressor cells; IP10, IFNγ-induced protein 10; FRα, folate receptor α; IL-15, interleukin-15; PD-L1, programmed cell death protein-ligand 1; CTLA-4, cytotoxic T-lymphocyte-associated protein 4 Tregs, T regulatory cells; CIK, cytokine induced killer cells; MG1-IL12-ICV, Maraba virus MG1 with interleukin 12-infected cell vaccine; IP, intraperitoneal; NKs, natural killer cells; IL-21, interleukin 21; MUC1, mucin1; MAGE-A1, melanoma associated antigen-A1; FBP, folate binding protein;.

**Table 3:** Trifunctional antibody for peritoneal metastasis.

Cancer Type	Treatment	Target	Model	Author (Year)
Gastric cancer	Intra and postoperatively administered Catumaxomab	EpCAM	Human	Bokemeyer et al. [88] (2015)
Colorectal cancer	Catumaxomab	EpCAM	Human	Borlak et al. [89] (2015)
Ovarian cancer	Catumaxomab	EpCAM	Human	Wimberger et al. [90] (2012)
Ovarian, pancreatic, colon, gastric, breast	Catumaxomab+paracentesis	EpCAM	Human	Heiss et al. [86] (2010)
Gastric, ovarian, PM ααom unknown primary	Catumaxomab	EpCAM	Human	StrÖhlein et al. [91] (2009)
Ovarian cancer	Catumaxomab	EpCAM	Human	Burges et al. [92] (2007)
Colon cancer	Catumaxomab	EpCAM	Murine	Ruf et al. [93] (2007)

Abbreviations: EpCAM, epithelial cell adhesion molecule.
