# Erratum: Thadi A.; et al. Early Investigations and Recent Advances in Intraperitoneal Immunotherapy for Peritoneal Metastasis. *Vaccines* 2018, *6*, 54

**DOI:** 10.3390/vaccines7010015

**Published:** 2019-01-31

**Authors:** Anusha Thadi, Marian Khalili, William F. Morano, Scott D. Richard, Steven C. Katz, Wilbur B. Bowne

**Affiliations:** 1 Department of Surgery, Drexel University College of Medicine, Philadelphia, PA 19102, USA; anusha.thadi@gmail.com (A.T.); mariankhalili@gmail.com (M.K.); morano.william@gmail.com (W.F.M.); 2 Department of Obstetrics and Gynecology, Thomas Jefferson University Hospital, Philadelphia, PA 19107, USA; scott.richard@jefferson.edu; 3 Department of Surgery, Boston University School of Medicine, Boston, MA 02118, USA; skatz@chartercare.org; 4 Roger Williams Medical Center, Providence, RI 02908, USA

The authors wish to make the following corrections to this paper [1]:

The 5th author, Steven C. Katz was affiliated with the Department of Surgery, Boston University School of Medicine, Boston, MA 02118, USA. The authors would like to add the following 4th affiliation to Steven C. Katz: “Roger Williams Medical Center, Providence, RI 02908, USA” 

The authors would like to apologize for any inconvenience caused to the readers by these changes.
